# Uses of augmented reality in surgical consent and patient education – A systematic review

**DOI:** 10.1371/journal.pdig.0000777

**Published:** 2025-04-28

**Authors:** Thomas Evans, Adam Turna, Thomas D. Stringfellow, Gareth G. Jones

**Affiliations:** 1 Trauma and Orthopaedic Registrar, Department of Trauma and Orthopaedics, Isle of Wight NHS Trust, Newport, United Kingdom; 2 Resident Doctor, Department of Trauma and Orthopaedics, East and North Hertfordshire NHS Trust, Stevenage, United Kingdom; 3 GIRFT Research Fellow and Trauma and Orthopaedic Registrar, Department of Trauma and Orthopaedics, Royal National Orthopaedic Hospital NHS Trust, London, United Kingdom; 4 Consultant Trauma and Orthopaedic Surgeon and Clinical Senior Lecturer in Orthopaedic Surgery, Department of Trauma and Orthopaedics, Imperial College NHS Foundation Trust and Imperial, London, United Kingdom; University of Pennsylvania, United States of America

## Abstract

Augmented reality (AR) allows the real environment to be altered with superimposed graphics using a head-mounted-display (HMD), smartphone or tablet. AR in surgery is being explored as a potential disruptive technology and could be used to improve patient understanding of treatment and as an adjunct for surgery. The aim was to explore this use of AR and assess potential benefits for consent and patient education. A systematic review was conducted using PRISMA-SCR guidelines. 4 major bibliographic databases were searched using the terms: ‘(augmented reality OR mixed reality) AND surgery AND (consent OR patient education)’. Included papers evaluated an AR intervention on consenting patients for enhancing surgical consent or education about a procedure. Non-English language papers and studies which did evaluate an intervention were excluded. Three reviewers screened all abstracts and full text papers for inclusion. The review protocol was prospectively registered with PROSPERO (ID: CRD42020207360). 52 records were identified. Following removal of 13 duplicates, 21 were removed after abstract screening leaving 17 articles for full assessment. One article was a letter and 8 did not evaluate interventions, leaving 8 articles published between 2019 and 2023. 3 papers were randomised controlled trials comparing AR enhanced processes to standard consent, 2 cohort studies evaluated patient satisfaction with AR interventions and there was one randomised crossover trial of AR against traditional consent consultation. The Cochrane risk of bias tool was used most studies were deemed as high risk of bias. Patient satisfaction and understanding were improved using AR. However, advantages over other enhanced techniques are less clear. Using AR to enhance written literature was shown to require less mental effort from patients and was preferred to standard resources to understand complex surgery. The few randomised trials are limited by bias and lack of power calculation, highlighting the need for further research.

## Author summary

This study used a robust evidence search to identify and synthesise key applications of augmented reality (AR) in the field of surgery. Augmented reality is a technique where graphics can be superimposed onto models or a real environment. Papers that attempted to improve patients’ understanding of surgical procedures and/ or better communicate risk of surgery using AR were included in the final review. There were 8 papers that met the criteria for our review published between 2000 and 2023 included in the review that examined interventions in a range of surgical specialties and used different study types. Most of the articles reported a benefit to using AR including better mental comprehension of complex concepts, visualisation of anatomical structures and better understanding of what a certain operation involves. Some of the studies included were prone to bias, specifically ‘novel technology’ bias whereby the benefits are likely over-stated by participants. We concluded that AR could improve patients’ understanding of difficult concepts which are important to convey during the surgical consent process. Higher quality evidence is needed in this field and it unclear to what extent AR can provide benefits over less expensive models and moving images.

## Introduction

### Augmented reality technology

Augmented reality (AR) is an evolving technology which allows three-dimensional computer-generated graphics to be superimposed onto physical objects in the real environment via the use of a viewing device [1]. Another term given to this technology is ‘mixed reality’ or ‘virtual environments’, which are perceived to be the future for innovation in a range of consumer, industrial and scientific settings [2]. The development of AR for use in surgery is in its infancy, however it is clear there may be potential benefits to its use for many aspects of surgical and clinical practice [3].

There are key differences between AR and virtual reality (VR). VR sees the user fully engaged in a virtual environment [4] and gives a ‘perception’ of being in this environment [2]. AR incorporates computer-generated elements with the real environment and physical objects, creating a fusion or blending of the real world and virtual elements [4]. A fundamental concept of AR is that of ‘registration’, this is how the real-world objects spatially connect to the superimposed data or graphics [5].This is typically done with a ‘reference frame’, which is a fixed point the computer uses to reference throughout the activity. Newer generation AR systems are finding ways to effectively remove the need for a reference marker.

There are four key components to an AR system: a computer, tablet or smartphone; a software graphics rendering package; a viewing device or electronic display – this may be a smartphone with camera or head-mounted display (HMD), usually an AR headset or smart-glasses – and a reference frame (physical object to track or mounted QR code, for example) [6]. There are a number of commercially available AR headsets, including Hololens by Microsoft, Google Glass by Google and Magic Leap 1 by Magic Leap.

There is also recognition of the need for quality input data to allow for the most accurate and clinically appropriate translation of data into AR for use in surgery [6]. Consideration of the limitations of AR in the early aspects of its use means there is a feeling that routine, commonplace use of AR in surgery is still a way off [7].

### Potential uses for AR technology in surgery

Uses for AR can be considered from both patient and clinician perspectives [4]. Its use can be divided into pre-operative, intra-operative and post-operative stages of the patient pathway, as well as its role in training surgeons and wider medical education [8,9]. Pre-operative uses include patient-specific surgical planning [10], patient education and use as a tool for visualisation and understanding of the procedure for consent [11]. Intra-operatively, potential roles for AR include visualisation of pre-operative imaging, fluoroscopy, navigation of complex anatomy and implant positioning [12]. Post-operative uses include rapid access to patient diagnostics, laboratory results, integration of further investigation results and vital signs in real time [13].

Uses for AR in training include rehearsal of operative or procedural steps and stages, remote assistance/guidance and immersive teaching programs [5,9]. There is also the role for AR in improving understanding of how to correct deformities or problems intraoperatively [5] and immersive learning of important and relevant anatomy [14]. It is not unrealistic to view AR in surgery as a potential ‘disruptive technology’, with Moro *et al.* [14] recognising adverse effects for users of AR and the need for additional personnel in technical setup/aspects of AR use in surgery [10]. However, as with all new techniques and technologies in surgery, each role must be robustly evaluated with high quality clinical research methods to assess benefits and drawbacks. There is currently no classification system for applications of AR in surgery.

One of the limitations of AR is the need for a physical reference marker to ensure intra-operative patient movement does not impact on the accuracy of the system. Marker-less systems have yet to prove as reliable and their use is currently limited even within pre-clinical research [5]. Another issue is accommodation and focusing conflict with HMDs, creating a useable overlay depth of field is important to ensure minimal visual strain when using AR systems intra-operatively, with headaches and blurred vision cited as recognised adverse effects for HMD users [14]. Furthermore, the HMD must be designed carefully to prevent problems with sterile field contamination, user fatigue and strain whilst allowing the surgeon to perform the procedure or task in the real environment with minimal interference when necessary. HMDs are the preferred AR devices in intra-operative applications due to their minimal invasion of the operative field and other user factors.

### Uses of AR in surgical consent and patient education

In the UK the cost of clinical negligence and impact of surgical complications is vast [15] and this is a potential area where AR use could be implemented. Several surgical specialties have examined AR’s potential benefits and limitations in assisting the surgical consent process. In the current medicolegal landscape, it is important clinicians find new ways to assist patients in making decisions around surgery and maximise the informed nature of the consent process [16].

The aim of this paper is to systematically analyse the literature for published work examining the role of AR in the surgical consent and patient education process, highlight potential benefits and limitations of this and identify areas for further work and future research.

## Methods

A search strategy was designed around PRISMA guidelines for scoping reviews [17]. This was PROSPERO registered in line with criteria for inclusion of systematic reviews (PROSPERO registration ID CRD42020207360). The use of AR for surgical consent is a novel area and a meta-analysis was not planned due to a likely paucity of evidence.

A literature search was performed of Medline, PubMed, EMBASE and Google Scholar bibliographic databases using the following search terms ‘(augmented reality OR mixed reality) AND surgery AND (consent OR patient education)’.

These terms were chosen to yield maximal results and identify papers that focused on the use of AR to improve patient understanding of complex concepts or procedures. Results were limited to papers published after the year 2000 in English language (or translated to English). The aim of this review was to evaluate the effectiveness of AR in consenting surgical patients and patient education interventions. Included papers evaluated an AR intervention on consenting patients for enhancing surgical consent or education about a procedure. Non-English language papers and studies which did evaluate an intervention were excluded.

**Fig 1:**
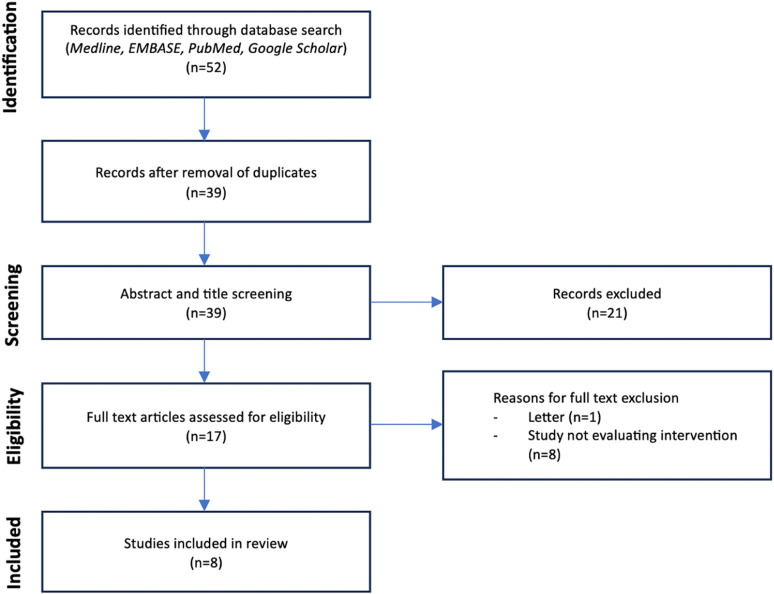
PRISMA Diagram for the systematic scoping review, search terms ‘(augmented reality OR mixed reality) AND surgery AND (consent OR patient education)’. No additional records were added after reference review of included studies.

All references were exported with their abstracts to a word processing document for each database searched. All abstracts and full text articles were screened independently by the reviewers (TDS, TE and GGJ). A fourth reviewer (AT) was used to adjudicate for any disagreements. Full text articles were read for each included study after abstract screening and reference lists for each were analysed for potential further studies to be included via abstract screening of relevant papers. Fig 1 outlines the systematic scoping methodology in line with PRISMA techniques. The original search was conducted in April 2020 with a further search in August 2020 to incorporate latest results. A further search was conducted with the same search terms in February 2023 on account of the paucity of available literature found in the initial search period. The same search criteria and methods were used in this additional round of searches.

## Results and discussion

The search yielded fifty-two results, of which 13 were removed due to duplication. A further 21 did not cover the inclusion criteria on review of the abstract. 8 papers were included following full text review by both authors (Fig 1).

**Fig 2:**
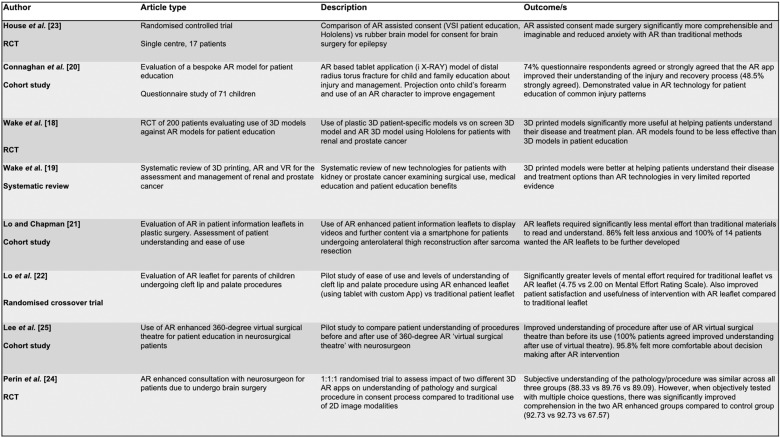
Summary of papers included in the final systematic review.

One of the full text papers was ultimately excluded due to being a published commentary style letter and another eight were excluded as they did not evaluate an intervention. Each paper included in the final study is summarised and discussed below and in Fig 2.

All included studies were published between 2019 and 2023. Two studies examined use of AR in urology [18,19], one study focussed on trauma and orthopaedics [20], two studies were in plastic and reconstructive surgery [21,22] and three studies were in neurosurgery [23,24,25]. The three randomised controlled trials (RCTs) identified [18,23,24] and the single randomised crossover trial are assessed for risk of bias below using the Cochrane risk of bias tool (RoB-2) by consensus between two reviewers. Participant level variables were not extracted in this largely narrative analysis. Owing to heterogeneity between studies and highly variable experimental designs a narrative synthesis was chosen.

In neurosurgery for refractory epilepsy, House *et al*. [23] evaluated a commercially available software package combined with Microsoft Hololens. This was shown to significantly improve patient comprehension and imagining (ability for a patient to imagine the steps involved in such an operation) and relieve anxiety before surgery. This seventeen patient study compared standard verbal consent with imaging review to use of plastic brain model and patient-specific AR brain model with superimposed MRI and CT scans [23]. The authors conclude that AR consent made surgery significantly more comprehensible and imaginable than standard consent processes and aided a reduction of pre-operative patient anxiety. Limitations of this study include risk of bias due to the ‘new technology effect’, low sample size and limitations establishing how much information was retained by patients. There was also a possibility of unrealistic expectations of the simplicity and ease of completing the procedure by viewing animations on the HMD. Interestingly, the same study did not show a statistically significant increase in comprehension, imagining or reduction in anxiety for patients’ relatives. Three of the authors of this manuscript declare conflicts of interest as advisor, chief executive office and employee of the parent company of the software solution tested.

Wake *et al.* [18] assessed patients undergoing surgery for renal and prostate cancer. Patients were consented with imaging alone, 3D printed patient-specific models, 3D models viewed with AR (Microsoft Hololens) or the same model viewed on a standard computer monitor. This randomised study of 200 patients showed 3D printed models performed significantly better than imaging alone for patients’ understanding of their disease and surgical treatment plan. The paper found 3D models performed significantly better than AR models for improving patients’ understanding of anatomy, disease process and comfort level with treatment plan. However, detailed analysis of arms of the trial revealed that only 25 patients were randomised to the AR group compared with 55 and 46 in the 3D printed and 3D model on computer groups. No detail was provided on the randomisation technique. The authors followed up with a systematic review examining the role of 3D printing and AR for patients with urological cancer concluding that these technologies could have an increasing role in the management of these patients [19]. Aside from their own data on AR discussed above, they found no further studies examining AR specifically for pre-operative patient education.

In trauma and orthopaedic surgery, Connaghan *et al.* [20] evaluated a bespoke AR application – *‘I X-RAY’* – a tablet-based AR application to improve children’s and parents’ understanding of a common paediatric orthopaedic injury, a distal radius torus fracture. This injury is commonly managed in a removable splint without the need for specialist follow up and provision of high-quality information and reassurance is crucial. The application uses AR to overlay graphics onto the child’s arm to demonstrate their injury and show them how the bone is likely to heal whilst explaining their treatment and recovery plan. Sixty-six fully completed questionnaires from children between the ages of 5 and 15 years who used ‘i X-RAY’ were included. 74% of children stated their understanding of the injury and recovery process was improved after using the AR app. 89% of children and their families agreed or strongly agreed this technology should be used more in school and hospital environments. Unfortunately, this study was not randomised and tested in a healthy trial group but does show promise for the use of AR technology.

Lo and Chapman [21] reported a global first using an AR enhanced patient information leaflet for reconstructive surgery using an anterolateral thigh flap following sarcoma resection. Patients use a smartphone app to scan photographs on the leaflet which ‘come to life’ with videos and further information. The AR leaflet was found to require significantly less mental effort to comprehend (1.57 vs 6.36 on Mental Effort Rating Scale) than traditional resources. 86% of the fourteen patients in the study stated they felt less anxious about surgery after using the AR intervention with all respondents feeling similar leaflets would be beneficial for patients and that further AR enhanced leaflets should be developed. This highlights patient support for similar resources and the potential accessibility of AR supported patient education and consent by using the patient’s own smartphone, reducing the high setup costs associated with VMDs yet retaining the benefits brought by AR in improving patient understanding of treatment and surgical procedures.

Lo *et al.* [22] further evaluated the benefits of AR enhanced patient leaflets for parents of children undergoing cleft lip or palate procedures. They similarly found that the role of AR saw significant improvements in multiple domains when compared to traditional patient leaflets. An AR enhanced leaflet was used in a randomised crossover trial as a pilot study for further trials on the use of AR in patient consent or education. The trial showed less mental effort required to understand resources when scrutinised on the Mental Effort Rating Scale (2.00 for AR leaflet vs 4.75 for traditional methods), which was the primary outcome measure of the trial pilot. There was significantly improved visual analogue scale (VAS) for AR leaflet (93.50) than traditional methods (54.50) and recognition that AR enhanced leaflets are more useful for patients than conventional leaflet types (Usefulness Scale for Patient Information Material scores of 74.08 compared to 49.42). Furthermore, the Instructional Materials Motivation Survey (IIMS) result suggested improved motivation for choosing to proceed with surgical intervention after the use of AR enhanced leaflet when compared to traditional patient leaflets (161.75 compared to 112.50).

Lee *et al.* [25] similarly ran a pilot study comparing neurosurgical patient understanding of procedures before and after an AR intervention involving a 360-degree virtual surgical theatre session with a neurosurgeon. Understanding of procedures was improved in all patients included in the pilot (19 patients strongly agreed, 5 patients agreed on intervention questionnaire) and there were reported levels of both improved understanding of procedures – 9.3 vs 6.8 on questionnaire scale of 1-10 of understanding of procedure. 95.8% patients felt their inclusion in decision making was improved as a result.

The use of two different 3D imaging AR platforms (Surgical Theater™ in group 1 and Vesalius™ in group 2) prior to craniotomy was favourable in improving understanding of both pathology and the surgical process when compared to the traditional use of 2D imaging in the pre-operative consent process in the SPLICE (Surgical Planning and Informed Consent) study by Perin *et al.* [24]. Their three-armed trial showed that whilst there was a similar subjective level of understanding of the surgical process (88.33; 89.76; 89.09 on ad-hoc questionnaire scale), objectively there was significant improvement in comprehension of diagnosis, therapeutic alternatives, tumour and intervention type in the AR enhanced arms of the trial compared to the control group, with scores of 92.73 for both AR arms and 67.57 in the control group on a pre-designed multiple-choice question test given after the consultation. Interestingly, Perin *et al*. [24] did not see any meaningful difference in the levels of pre-procedure anxiety across the trial groups.

### Risk of bias of included trials

**Fig 3:**
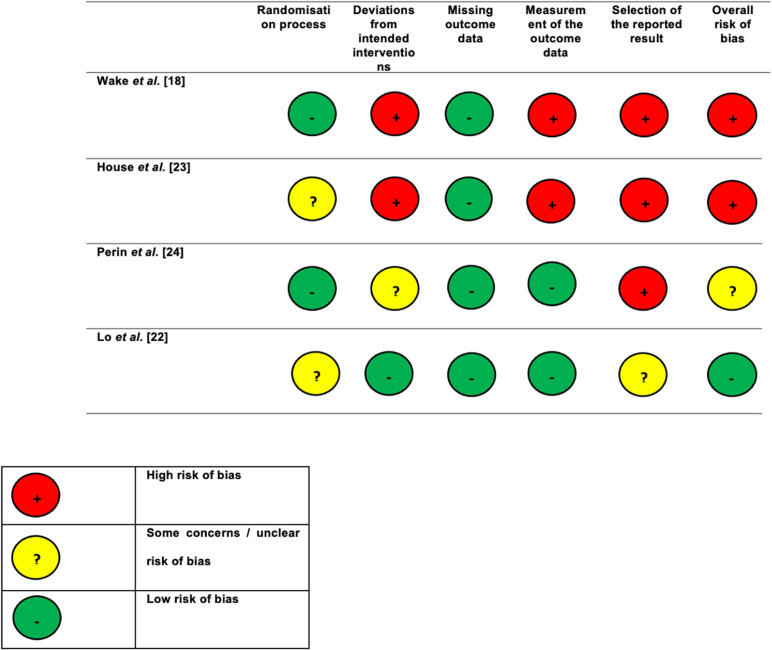
Cochrane risk of bias analysis for the intervention studies included in the review.

As previously mentioned, there was a significant risk of bias in the three RCTs included in this systematic review. Fig 3 demonstrates the extent of these risks using the Cochrane risk of bias toolkit (RoB-2).

The intervention trials included following the systematic literature search were found to have high risk of bias or some concern of bias risk. Neither House *et al.* [23] or Wake *et al.* [18] included power calculations in their methodology and the House *et al*. [23] study may have been conflicted by commercial interest. It was also likely to have been significantly underpowered. Overall, the risk of bias in these papers was significant. Perin *et al.’s* [24] SPLICE study was slightly more robust in its methodology and returned some concern of bias when calculated through the RoB-2 toolkit. Lo *et al.*’s [22] randomised crossover study was also analysed for risk of bias and showed low risk of bias according to the calculations. However, it is worth pointing out that this was a pilot study with a small study group and so the significance of this low-risk status must be taken in the context of these details.

Overall, the nature of the trials analysed in this review means that a true blinding process is very difficult. By analysing the effects of AR in consent and education processes, being able to conceal the arm of the trial each participant is in would realistically be impossible. However, small sample sizes and other external risks mean that the potential risk of bias for the studies is nonetheless still high.

Literature reports of applications of augmented reality in this field should describe the development of the product and involve patient or carer reported measures of effectiveness and include a comparator group where possible as well as mental effort scores to assess the ease of understanding. This could follow healthcare technology assessment methodology. Simpler and cheaper uses of AR are likely to be easier to deploy in healthcare systems where hardware purchasing can be prohibitive. Applications could be classified according to level of fidelity from simple enhanced written materials viewed using a smartphone through to high-fidelity smart glasses programmes for patients. Classification could improve searchability of reports and provide a common language similar to that for medical simulation.

### Limitations

The use of AR in patient education and surgical consenting process is a poorly studied application of the technology and there is little high-quality evidence available to analyse. The trials which have attempted to randomise patients to standard and AR enhanced consent pathways for surgery give conflicting conclusions to the value of AR for patient education and there are issues surrounding risks of bias and/or conflict of interest. Further randomised controlled trials are required in this area and would ultimately answer the question of how this technology could enhance and improve the surgical consent process for patients. Whilst the authors of this systematic review are confident the literature search strategy was carefully constructed to yield maximum relevant results, it is possible some records in grey literature or conference proceedings were not detected and given the rapidly evolving nature of technology in surgery there is a real possibility that more current literature has since been published.

## Conclusion

There are few published studies demonstrating the use of AR for surgical consent and patient education processes and most of the evidence is descriptive and experimental in nature. Grouped analyses of the current literature is not possible. Studies detailed in this paper where AR consent interventions are evaluated are limited by small sample sizes, unequal subgroup allocation and selection bias. Despite this, patients in almost all the studies preferred an AR enhanced consent or information programme compared to standard or traditional resources hence proving a foundation for these technologies [20,21,23,25].

There is certainly potential for the use of AR in improving surgical patient education and it is likely, especially in children, that using this technology could improve engagement and potentially aid retention of more information about their condition and the full scope of treatment [20]. Barriers to the technology in a clinical setting have not been fully explored nor has the ability of elderly patient groups to engage with AR. Surgical practice encompasses patients of all ages and it is important the needs of all patient groups are considered with any new intervention. There may be particular value in utilising AR to help explain concepts that cannot be easily relayed to patients with simple 2D imaging or where particularly complex elements are being explained [20,21,23].

There is currently a lack of well-designed RCTs within the literature to evaluate AR in the surgical consent and patient education process. The benefits of AR over other technologies such as 3D printed models and 3D models viewed on computers or portable devices must be carefully assessed [19]. Power calculations assessing interventions are needed and more robust efforts to control the effect of performance and detection bias are required. The expensive nature of AR and emerging technologies means a strong evidence base needs to be established before routinely utilising AR software packages and hardware in publicly funded healthcare systems to ensure good value for money and reasonable use and distribution of valuable resources. The use of artificial intelligence (AI) – a topical and similarly emerging technology- to enhance written information and patient communications is another potential solution that could improve patient education whilst delivering value for money [21].

Overall, the evidence presented suggests patients tend to prefer information regarding their condition and surgery using AR enhanced resources and their understanding may be improved as a result. This paper provides a foundation for the ongoing work in the area and support for further related research.

## Take home messages

- Most studies showed AR increased patients’ understanding of their disease process and proposed treatments above standard discussion and imaging review
- AR may be of most value for complex procedures or where there are complex decisions regarding treatment options
- Further high-quality and carefully designed trials would help assess benefits of AR for this specific use
- Studies on accessibility and ease of use of AR technology for different patient demographics are required
- AR enhanced patient information leaflets are a low-cost intervention which may deliver the added benefits of AR by using patients’ own smartphones or tablets

## Supporting information

S1 File Data from systematic review in file named ‘records after title and abstract screening’.
(DOCX)
