# Supplementary material for: Uses of augmented reality in surgical consent and patient education – A systematic review
Source: PLOS Digit Health. 2025 Apr 28;4(4):e0000777. doi: 10.1371/journal.pdig.0000777 (PMC12036893; doi:10.1371/journal.pdig.0000777)
Supplement: S1 File — Data from systematic review in file named ‘records after title and abstract screening’. (DOCX) [file pdig.0000777.s001.docx]

1. **3D Printing, Augmented Reality, and Virtual Reality for the Assessment and Management of Kidney and Prostate Cancer: A Systematic Review. [Review]**

Wake N; Nussbaum JE; Elias MI; Nikas CV; Bjurlin MA.

Ovid MEDLINE(R) ALL

Urology. 2020 Jun 12.

[Journal Article. Review]

UI: 32535076

Three-dimensional (3D) printing, augmented reality, and virtual reality technologies have an increasing presence in the management of prostate and kidney cancer. To assess the utility of 3D printing, augmented reality, and virtual reality for (1) quantitative outcomes, (2) surgical planning, (3) intraoperative guidance, (4) training and simulation, and (5) patient education for patients with kidney and prostate cancer a systematic literature review was performed. Existing evidence demonstrates improvement in clinical outcomes, surgical planning and intra-operative guidance, as well as training. Future studies are needed to assess the impact of 3D technologies on long-term patient-related outcomes.

Copyright © 2020 Elsevier Inc. All rights reserved.

Version ID

1

Record Owner

From MEDLINE, a database of the U.S. National Library of Medicine.

Status

Publisher

Authors Full Name

Wake, Nicole; Nussbaum, Jeffrey E; Elias, Marie I; Nikas, Christine V; Bjurlin, Marc A.

Institution

Wake, Nicole. Department of Radiology, Montefiore Medical Center, Albert Einstein College of Medicine, Bronx, NY; Center for Advanced Imaging Innovation and Research (CAI2R) and Bernard and Irene Schwartz Center for Biomedical Imaging, Department of Radiology, NYU Langone Health, NYU School of Medicine, New York, NY. Electronic address: nwake@montefiore.org. Nussbaum, Jeffrey E. Department of Radiology, Montefiore Medical Center, Albert Einstein College of Medicine, Bronx, NY.

Elias, Marie I. Health Sciences Library, Montefiore Medical Center, Bronx, NY.

Nikas, Christine V. Department of Urology, University of North Carolina, NC.

Bjurlin, Marc A. Department of Urology, University of North Carolina, NC.

Year of Publication

2020

Link to the Ovid Full Text or citation:

[Click here for full text options](https://ovidsp.ovid.com/athens?T=JS&CSC=Y&NEWS=N&PAGE=fulltext&D=medp&AN=32535076)

Link to the External Link Resolver:

[SFX](http://sfxeu05.hosted.exlibrisgroup.com/44RCS?sid=OVID:medline&id=pmid:32535076&id=doi:10.1016%2Fj.urology.2020.03.066&issn=0090-4295&isbn=&volume=&issue=&spage=&pages=&date=2020&title=Urology&atitle=3D+Printing%2C+Augmented+Reality%2C+and+Virtual+Reality+for+the+Assessment+and+Management+of+Kidney+and+Prostate+Cancer%3A+A+Systematic+Review.&aulast=Wake&pid=%3Cauthor%3EWake+N%3BNussbaum+JE%3BElias+MI%3BNikas+CV%3BBjurlin+MA%3C%2Fauthor%3E%3CAN%3E32535076%3C%2FAN%3E%3CDT%3EJournal+Article%3C%2FDT%3E)

2. **Use of the mixed reality tool "VSI Patient Education" for more comprehensible and imaginable patient educations before epilepsy surgery and stereotactic implantation of DBS or stereo-EEG electrodes.**

House PM; Pelzl S; Furrer S; Lanz M; Simova O; Voges B; Stodieck SRG; Bruckner KE.

Ovid MEDLINE(R) ALL

Epilepsy Research. 159:106247, 2020 01.

[Journal Article]

UI: 31794952

PURPOSE: It is unknown which patient education strategy before epilepsy surgery or stereotactic electrode implantation is best for patients. This prospective and randomized clinical study investigates whether the use of the mixed reality tool "VSI Patient Education" (VSI PE) running on HoloLens R glasses is superior to the use of a rubber brain model as a 3-dimensional tool for patient education before epilepsy surgery and stereotactic electrode implantation.

MATERIAL AND METHODS: 17 patients with indication for epilepsy surgery or stereotactic electrode implantation were included in the study and randomized into two groups. All patients were informed with both comparative tools VSI PE (apoQlar R) and a rubber brain model (3B Scientific R) in a chronological order depending on group assignment. Afterwards, the patient and, if present, a relative (12) each filled out a questionnaire. For statistical analysis, Wilcoxon rank-sum tests were performed.

RESULTS: Patients found their patient education highly significantly more comprehensible (p = 0.001**, r = 0.84) and almost significantly more imaginable (p=0.020, r = 0.57), when their doctor used VSI PE compared to the rubber brain model. The patients felt significantly less anxious as a result of VSI PE (p = 0.008*, r = 0.64). Highly significantly more patients chose VSI PE as the preferred patient education tool (p < 0.001**, r = 0.91), and almost significantly more patients decided VSI PE to be the future standard tool (p = 0.020, r = 0.56). Significantly more relatives chose VSI PE as the preferred patient education tool (p = 0.004*, r = 0.83), and significantly more relatives decided VSI PE to be the future standard tool (p = 0.002*, r = 0.91).

CONCLUSION: VSI Patient Education is a promising new mixed reality tool for informing patients before epileptic surgery or stereotactic electrode implantation in order to enhance comprehension and imagination and reduce fear and worries. It might strengthen patient commitment and have a positive influence on patients' decisions in favor of medically indicated surgical operations.

Copyright © 2019 Elsevier B.V. All rights reserved.

Version ID

1

Record Owner

From MEDLINE, a database of the U.S. National Library of Medicine.

Status

In-Process

Authors Full Name

House, Patrick M; Pelzl, Sirko; Furrer, Simon; Lanz, Michael; Simova, Olga; Voges, Berthold; Stodieck, Stefan R G; Bruckner, Katja E.

Institution

House, Patrick M. Hamburg Epilepsy Center, Protestant Hospital Alsterdorf, Department of Neurology and Epileptology, Hamburg, Germany. Electronic address: p.house@eka.alsterdorf.de. Pelzl, Sirko. apoQlar GmbH, Hamburg, Germany.

Furrer, Simon. apoQlar GmbH, Hamburg, Germany.

Lanz, Michael. Hamburg Epilepsy Center, Protestant Hospital Alsterdorf, Department of Neurology and Epileptology, Hamburg, Germany.

Simova, Olga. Hamburg Epilepsy Center, Protestant Hospital Alsterdorf, Department of Neurology and Epileptology, Hamburg, Germany.

Voges, Berthold. Hamburg Epilepsy Center, Protestant Hospital Alsterdorf, Department of Neurology and Epileptology, Hamburg, Germany.

Stodieck, Stefan R G. Hamburg Epilepsy Center, Protestant Hospital Alsterdorf, Department of Neurology and Epileptology, Hamburg, Germany.

Bruckner, Katja E. Hamburg Epilepsy Center, Protestant Hospital Alsterdorf, Department of Neurology and Epileptology, Hamburg, Germany.

Keyword Heading

*3D *HoloLens

*MRI

*Mixed reality

*Patient education

*VSI.

Year of Publication

2020

Link to the Ovid Full Text or citation:

[Click here for full text options](https://ovidsp.ovid.com/athens?T=JS&CSC=Y&NEWS=N&PAGE=fulltext&D=prem&AN=31794952)

Link to the External Link Resolver:

[SFX](http://sfxeu05.hosted.exlibrisgroup.com/44RCS?sid=OVID:medline&id=pmid:31794952&id=doi:10.1016%2Fj.eplepsyres.2019.106247&issn=0920-1211&isbn=&volume=159&issue=&spage=106247&pages=106247&date=2020&title=Epilepsy+Research&atitle=Use+of+the+mixed+reality+tool+%22VSI+Patient+Education%22+for+more+comprehensible+and+imaginable+patient+educations+before+epilepsy+surgery+and+stereotactic+implantation+of+DBS+or+stereo-EEG+electrodes.&aulast=House&pid=%3Cauthor%3EHouse+PM%3BPelzl+S%3BFurrer+S%3BLanz+M%3BSimova+O%3BVoges+B%3BStodieck+SRG%3BBruckner+KE%3C%2Fauthor%3E%3CAN%3E31794952%3C%2FAN%3E%3CDT%3EJournal+Article%3C%2FDT%3E)

3. Augmented reality in dermatology: Are we ready for AR?.

Sharma P; Vleugels RA; Nambudiri VE.

Ovid MEDLINE(R) ALL

Journal of the American Academy of Dermatology. 81(5):1216-1222, 2019 Nov.

[Journal Article]

UI: 31302186

Augmented reality (AR) refers to a group of technologies that capture, analyze, and superimpose digital information onto the real world. This information gives health care providers unique and useful perspectives that can enhance patient care. AR has been utilized in selected scenarios in health care for several decades, notably laparoscopic surgery and vein finding. In recent years, improved wireless technologies, computing power, and analytics are leading to rapid growth in the AR industry. Novel health care-specific use cases are rapidly being introduced with the potential to widely affect clinical care, particularly in dermatology because of the visual nature of the field. In this article, we define AR, profile clinical and educational uses of AR in dermatology, and discuss key policy considerations for the safe and appropriate use of this emerging technology.

Copyright © 2019 American Academy of Dermatology, Inc. Published by Elsevier Inc. All rights reserved.

Version ID

1

Record Owner

From MEDLINE, a database of the U.S. National Library of Medicine.

Status

MEDLINE

Authors Full Name

Sharma, Priyank; Vleugels, Ruth Ann; Nambudiri, Vinod E.

Institution

Sharma, Priyank. Department of Dermatology, Brigham and Women's Hospital, Boston, Massachusetts. Vleugels, Ruth Ann. Department of Dermatology, Brigham and Women's Hospital, Boston, Massachusetts.

Nambudiri, Vinod E. Department of Dermatology, Brigham and Women's Hospital, Boston, Massachusetts. Electronic address: vnambudiri@bwh.harvard.edu.

MeSH Heading

*Augmented Reality. Dermatology/ec [Economics]. *Dermatology/mt [Methods]. Humans. *Skin Diseases/di [Diagnosis]. *Skin Diseases/th [Therapy].

Keyword Heading

augmented reality dermatology

digital imaging

health policy

innovation

lesion measurement

lesion tracking

patient education

technology

virtual reality.

Year of Publication

2019

Link to the Ovid Full Text or citation:

[Click here for full text options](https://ovidsp.ovid.com/athens?T=JS&CSC=Y&NEWS=N&PAGE=fulltext&D=medl&AN=31302186)

Link to the External Link Resolver:

[SFX](http://sfxeu05.hosted.exlibrisgroup.com/44RCS?sid=OVID:medline&id=pmid:31302186&id=doi:10.1016%2Fj.jaad.2019.07.008&issn=0190-9622&isbn=&volume=81&issue=5&spage=1216&pages=1216-1222&date=2019&title=Journal+of+the+American+Academy+of+Dermatology&atitle=Augmented+reality+in+dermatology%3A+Are+we+ready+for+AR%3F.&aulast=Sharma&pid=%3Cauthor%3ESharma+P%3BVleugels+RA%3BNambudiri+VE%3C%2Fauthor%3E%3CAN%3E31302186%3C%2FAN%3E%3CDT%3EJournal+Article%3C%2FDT%3E)

4. Evaluation of Child-Friendly Augmented Reality Tool for Patient-Centered Education in Radiology and Bone Reconstruction.

Connaghan R; Poyade M; Rea PM.

Ovid MEDLINE(R) ALL

Advances in Experimental Medicine & Biology. 1171:105-126, 2019.

[Journal Article]

UI: 31823243

The use of augmented reality (AR) has a rich history and is used in a number of fields. Its application in healthcare and anatomy education is developing considerable interest. However, although its popularity is on the rise, its use as an educational and practical tool has not been sufficiently evaluated, especially with children. Therefore, this study presents the design, development and evaluation of an educational tablet-based application with AR functionality for children. A distal radius fracture was chosen, as it is one of the more common fractures in the younger age group. Following a standardized software engineering methodology, we identified functional and non-functional requirements, creating a child-friendly tablet based AR application. This used industry standard software and incorporated three-dimensional models of a buckle fracture, object and image target marker recognition, interactivity and educational elements. In addition, we surveyed children at the Glasgow Science Centre on its usability, design and educational effectiveness. Seventy-one children completed a questionnaire (25 also underwent a short structured interview). Overall, the feedback was positive relating to entertainment value, graphic design, usability and educational scope of the application. Notably, it was shown to increase user understanding of radiology across all age groups following a trial of the application. This study shows the great potential of using digital technologies, and more particularly augmented information, in engaging future generations in science from a young age. Creation of educational materials using digital technologies, and evaluating its effectiveness, highlights the great scope novel technology could have in anatomical education and training.

Version ID

1

Record Owner

From MEDLINE, a database of the U.S. National Library of Medicine.

Status

MEDLINE

Authors Full Name

Connaghan, Ruth; Poyade, Matthieu; Rea, Paul M.

Institution

Connaghan, Ruth. Anatomy Facility, School of Life Sciences, College of Medical, Veterinary and Life Sciences, University of Glasgow, Glasgow, UK. Connaghan, Ruth. School of Simulation and Visualization, The Glasgow School of Art, Glasgow, UK.

Connaghan, Ruth. Touch Surgery, London, UK.

Poyade, Matthieu. School of Simulation and Visualization, The Glasgow School of Art, Glasgow, UK.

Rea, Paul M. Anatomy Facility, Thomson Building, School of Life Sciences, College of Medical, Veterinary and Life Sciences, University of Glasgow, Glasgow, UK. Paul.Rea@glasgow.ac.uk.

MeSH Heading

*Augmented Reality. Child. Humans. Patient Education as Topic/st [Standards]. *Patient Education as Topic. Patient-Centered Care. Scotland. Software/st [Standards]. Surveys and Questionnaires. *User-Computer Interface.

Keyword Heading

3-D imaging techniques Anatomy teaching

Augmented reality

Digital anatomy

Education

Paediatrics.

Year of Publication

2019

Link to the Ovid Full Text or citation:

[Click here for full text options](https://ovidsp.ovid.com/athens?T=JS&CSC=Y&NEWS=N&PAGE=fulltext&D=medl&AN=31823243)

Link to the External Link Resolver:

[SFX](http://sfxeu05.hosted.exlibrisgroup.com/44RCS?sid=OVID:medline&id=pmid:31823243&id=doi:10.1007%2F978-3-030-24281-7_9&issn=0065-2598&isbn=&volume=1171&issue=&spage=105&pages=105-126&date=2019&title=Advances+in+Experimental+Medicine+%26+Biology&atitle=Evaluation+of+Child-Friendly+Augmented+Reality+Tool+for+Patient-Centered+Education+in+Radiology+and+Bone+Reconstruction.&aulast=Connaghan&pid=%3Cauthor%3EConnaghan+R%3BPoyade+M%3BRea+PM%3C%2Fauthor%3E%3CAN%3E31823243%3C%2FAN%3E%3CDT%3EJournal+Article%3C%2FDT%3E)

5. Is Reality Limiting Patient Understanding? A Discussion of the Implications of Augmented Reality Technology to Patient Understanding.

Zucker BE; Tekkis P; Kontovounisios C.

Ovid MEDLINE(R) ALL

Surgical Innovation. 25(2):188-189, 2018 04.

[Letter]

UI: 29303060

Version ID

1

Record Owner

From MEDLINE, a database of the U.S. National Library of Medicine.

Status

MEDLINE

Author NameID

Kontovounisios, Christos; ORCID: <https://orcid.org/0000-0002-1828-1395>

Authors Full Name

Zucker, Benjamin E; Tekkis, Paris; Kontovounisios, Christos.

Institution

Zucker, Benjamin E. 1 Imperial College London, London, UK. Tekkis, Paris. 1 Imperial College London, London, UK.

Tekkis, Paris. 2 Chelsea and Westminster Hospital, London, UK.

Kontovounisios, Christos. 1 Imperial College London, London, UK.

Kontovounisios, Christos. 2 Chelsea and Westminster Hospital, London, UK.

MeSH Heading

*Communication. Health Knowledge, Attitudes, Practice. Humans. *Patient Education as Topic. *Physician-Patient Relations. *Virtual Reality.

Year of Publication

2018

Link to the Ovid Full Text or citation:

[Click here for full text options](https://ovidsp.ovid.com/athens?T=JS&CSC=Y&NEWS=N&PAGE=fulltext&D=med15&AN=29303060)

Link to the External Link Resolver:

[SFX](http://sfxeu05.hosted.exlibrisgroup.com/44RCS?sid=OVID:medline&id=pmid:29303060&id=doi:10.1177%2F1553350617751462&issn=1553-3506&isbn=&volume=25&issue=2&spage=188&pages=188-189&date=2018&title=Surgical+Innovation&atitle=Is+Reality+Limiting+Patient+Understanding%3F+A+Discussion+of+the+Implications+of+Augmented+Reality+Technology+to+Patient+Understanding.&aulast=Zucker&pid=%3Cauthor%3EZucker+BE%3BTekkis+P%3BKontovounisios+C%3C%2Fauthor%3E%3CAN%3E29303060%3C%2FAN%3E%3CDT%3ELetter%3C%2FDT%3E)

6. Patient-specific 3D printed and augmented reality kidney and prostate cancer models: impact on patient education.

Wake N; Rosenkrantz AB; Huang R; Park KU; Wysock JS; Taneja SS; Huang WC; Sodickson DK; Chandarana H.

Ovid MEDLINE(R) ALL

3D Printing In Medicine. 5(1):4, 2019 Feb 19.

[Journal Article]

UI: 30783869

BACKGROUND: Patient-specific 3D models are being used increasingly in medicine for many applications including surgical planning, procedure rehearsal, trainee education, and patient education. To date, experiences on the use of 3D models to facilitate patient understanding of their disease and surgical plan are limited. The purpose of this study was to investigate in the context of renal and prostate cancer the impact of using 3D printed and augmented reality models for patient education.

METHODS: Patients with MRI-visible prostate cancer undergoing either robotic assisted radical prostatectomy or focal ablative therapy or patients with renal masses undergoing partial nephrectomy were prospectively enrolled in this IRB approved study (n = 200). Patients underwent routine clinical imaging protocols and were randomized to receive pre-operative planning with imaging alone or imaging plus a patient-specific 3D model which was either 3D printed, visualized in AR, or viewed in 3D on a 2D computer monitor. 3D uro-oncologic models were created from the medical imaging data. A 5-point Likert scale survey was administered to patients prior to the surgical procedure to determine understanding of the cancer and treatment plan. If randomized to receive a pre-operative 3D model, the survey was completed twice, before and after viewing the 3D model. In addition, the cohort that received 3D models completed additional questions to compare usefulness of the different forms of visualization of the 3D models. Survey responses for each of the 3D model groups were compared using the Mann-Whitney and Wilcoxan rank-sum tests.

RESULTS: All 200 patients completed the survey after reviewing their cases with their surgeons using imaging only. 127 patients completed the 5-point Likert scale survey regarding understanding of disease and surgical procedure twice, once with imaging and again after reviewing imaging plus a 3D model. Patients had a greater understanding using 3D printed models versus imaging for all measures including comprehension of disease, cancer size, cancer location, treatment plan, and the comfort level regarding the treatment plan (range 4.60-4.78/5 vs. 4.06-4.49/5, p < 0.05).

CONCLUSIONS: All types of patient-specific 3D models were reported to be valuable for patient education. Out of the three advanced imaging methods, the 3D printed models helped patients to have the greatest understanding of their anatomy, disease, tumor characteristics, and surgical procedure.

Version ID

1

Record Owner

From MEDLINE, a database of the U.S. National Library of Medicine.

Status

PubMed-not-MEDLINE

Author NameID

Wake, Nicole; ORCID: <http://orcid.org/0000-0002-8441-6059>

Authors Full Name

Wake, Nicole; Rosenkrantz, Andrew B; Huang, Richard; Park, Katalina U; Wysock, James S; Taneja, Samir S; Huang, William C; Sodickson, Daniel K; Chandarana, Hersh.

Institution

Wake, Nicole. Center for Advanced Imaging Innovation and Research (CAI2R) and Bernard and Irene Schwartz Center for Biomedical Imaging, Department of Radiology, NYU Langone Health, NYU School of Medicine, 660 First Avenue, Fourth Floor, New York, NY, 10016, USA. nicole.wake@nyulangone.org. Rosenkrantz, Andrew B. Center for Advanced Imaging Innovation and Research (CAI2R) and Bernard and Irene Schwartz Center for Biomedical Imaging, Department of Radiology, NYU Langone Health, NYU School of Medicine, 660 First Avenue, Fourth Floor, New York, NY, 10016, USA.

Huang, Richard. Division of Urologic Oncology, Department of Urology, NYU Langone Health, NYU School of Medicine, New York, NY, USA.

Park, Katalina U. Center for Advanced Imaging Innovation and Research (CAI2R) and Bernard and Irene Schwartz Center for Biomedical Imaging, Department of Radiology, NYU Langone Health, NYU School of Medicine, 660 First Avenue, Fourth Floor, New York, NY, 10016, USA.

Wysock, James S. Division of Urologic Oncology, Department of Urology, NYU Langone Health, NYU School of Medicine, New York, NY, USA.

Taneja, Samir S. Division of Urologic Oncology, Department of Urology, NYU Langone Health, NYU School of Medicine, New York, NY, USA.

Huang, William C. Division of Urologic Oncology, Department of Urology, NYU Langone Health, NYU School of Medicine, New York, NY, USA.

Sodickson, Daniel K. Center for Advanced Imaging Innovation and Research (CAI2R) and Bernard and Irene Schwartz Center for Biomedical Imaging, Department of Radiology, NYU Langone Health, NYU School of Medicine, 660 First Avenue, Fourth Floor, New York, NY, 10016, USA.

Chandarana, Hersh. Center for Advanced Imaging Innovation and Research (CAI2R) and Bernard and Irene Schwartz Center for Biomedical Imaging, Department of Radiology, NYU Langone Health, NYU School of Medicine, 660 First Avenue, Fourth Floor, New York, NY, 10016, USA.

PMC Identifier

<https://www.ncbi.nlm.nih.gov/pmc/articles/PMC6743040>

Keyword Heading

3D printing Augmented reality

Kidney cancer

Prostate cancer

Urologic oncology.

Year of Publication

2019

Link to the Ovid Full Text or citation:

[Click here for full text options](https://ovidsp.ovid.com/athens?T=JS&CSC=Y&NEWS=N&PAGE=fulltext&D=prem3&AN=30783869)

Link to the External Link Resolver:

[SFX](http://sfxeu05.hosted.exlibrisgroup.com/44RCS?sid=OVID:medline&id=pmid:30783869&id=doi:10.1186%2Fs41205-019-0041-3&issn=2365-6271&isbn=&volume=5&issue=1&spage=4&pages=4&date=2019&title=3D+Printing+In+Medicine&atitle=Patient-specific+3D+printed+and+augmented+reality+kidney+and+prostate+cancer+models%3A+impact+on+patient+education.&aulast=Wake&pid=%3Cauthor%3EWake+N%3BRosenkrantz+AB%3BHuang+R%3BPark+KU%3BWysock+JS%3BTaneja+SS%3BHuang+WC%3BSodickson+DK%3BChandarana+H%3C%2Fauthor%3E%3CAN%3E30783869%3C%2FAN%3E%3CDT%3EJournal+Article%3C%2FDT%3E)

7. The first worldwide use and evaluation of augmented reality (AR) in "Patient information leaflets" in plastic surgery.

Lo S., Chapman P.

Embase

Journal of Plastic, Reconstructive and Aesthetic Surgery. 73 (7) (pp 1357-1404), 2020. Date of Publication: July 2020.

[Letter]

AN: 2005613338

PMC Identifier

32317231 [<http://www.ncbi.nlm.nih.gov/pubmed/?term=32317231>]

Status

Embase

Author NameID

Lo, Steven; ORCID: <http://orcid.org/0000-0003-1503-1056>

Institution

(Lo) Canniesburn Plastic Surgery Unit, Glasgow Royal Infirmary, Glasgow G4 0SF, United Kingdom (Lo) College of Medical, Veterinary and Life Sciences, University of Glasgow, United Kingdom

(Lo) Translational Research Centre, Kaohsiung Medical University, Kaohsiung, Taiwan (Republic of China)

(Chapman) School of Simulation and Visualisation, The Glasgow School of Art, United Kingdom

Publisher

Churchill Livingstone

MeSH Heading

anterolateral thigh flap; *augmented reality; cancer surgery; game; *health literacy; hospital patient; human; letter; motivation; *patient education; patient satisfaction; *plastic surgery; priority journal; rating scale; sarcoma/su [Surgery]; leg sarcoma/su [Surgery]; mental effort rating scale.

Candidate Terms

leg sarcoma / surgery [other term]; mental effort rating scale [other term].

Official Date

anterolateral thigh flap; *augmented reality; cancer surgery; game; *health literacy; hospital patient; human; Letter; motivation; *patient education; patient satisfaction; *plastic surgery; priority journal; rating scale; sarcoma / surgery.

Year of Publication

2020

Link to the Ovid Full Text or citation:

[Click here for full text options](https://ovidsp.ovid.com/athens?T=JS&CSC=Y&NEWS=N&PAGE=fulltext&D=emexb&AN=2005613338)

Link to the External Link Resolver:

[SFX](http://sfxeu05.hosted.exlibrisgroup.com/44RCS?sid=OVID:embase&id=pmid:32317231&id=doi:10.1016%2Fj.bjps.2020.03.011&issn=1748-6815&isbn=&volume=73&issue=7&spage=1357&pages=1357-1404&date=2020&title=Journal+of+Plastic%2C+Reconstructive+and+Aesthetic+Surgery&atitle=The+first+worldwide+use+and+evaluation+of+augmented+reality+%28AR%29+in+%22Patient+information+leaflets%22+in+plastic+surgery&aulast=Lo&pid=%3Cauthor%3ELo+S.%3BChapman+P.%3C%2Fauthor%3E%3CAN%3E2005613338%3C%2FAN%3E%3CDT%3ELetter%3C%2FDT%3E)

8. Virtual reality in pediatric cardiology: Hype or hope for the future?.

Sacks L.D., Axelrod D.M.

Embase

Current Opinion in Cardiology. 35 (1) (pp 37-41), 2020. Date of Publication: 01 Jan 2020.

[Review]

AN: 630450871

Purpose of reviewIn the field of pediatric cardiology, a sub-specialty that relies on an understanding of complex three-dimensional structures, virtual reality technology may represent a new and exciting tool for both practitioners and patients. Here, the current medical and surgical applications of virtual reality are reviewed and the potential for future applications explored.Recent findingsMultiple centers have begun to develop software designed to bring virtual reality technology to bear on congenital heart disease. These efforts have focused on surgical preparation, on education of medical providers, and on preparation of patients and their family members for cardiac interventions. Though relatively little statistical evidence for benefit has been generated to date, those studies currently available suggest that virtual reality may provide a significant benefit in all three of these aspects of medical care.SummaryThe immersive nature of virtual reality meshes well with the learning styles of adult practitioners and promises to be a powerful tool for both pediatric cardiologists and the patients with whose care they are entrusted. Though additional studies are clearly needed, this technology promises to improve the field's ability to prepare providers and patients alike for challenge of treating congenital heart disease.

Copyright © 2019 Wolters Kluwer Health, Inc. All rights reserved.

PMC Identifier

31644471 [<http://www.ncbi.nlm.nih.gov/pubmed/?term=31644471>]

Status

Embase

Institution

(Sacks, Axelrod) Pediatric Cardiology, Stanford University School of Medicine, 750 Welch Road, Suite 321, Palo Alto, CA 94304, United States

Publisher

Lippincott Williams and Wilkins (E-mail: kathiest.clai@apta.org)

Keyword Heading

augmented reality, congenital heart disease, pediatric cardiology, virtual reality

MeSH Heading

cost benefit analysis; human; patient education; *pediatric cardiology; priority journal; review; *virtual reality.

Official Date

cost benefit analysis; human; patient education; *pediatric cardiology; priority journal; Review; *virtual reality.

Year of Publication

2020

Link to the Ovid Full Text or citation:

[Click here for full text options](https://ovidsp.ovid.com/athens?T=JS&CSC=Y&NEWS=N&PAGE=fulltext&D=emexa&AN=630450871)

Link to the External Link Resolver:

[SFX](http://sfxeu05.hosted.exlibrisgroup.com/44RCS?sid=OVID:embase&id=pmid:31644471&id=doi:10.1097%2FHCO.0000000000000694&issn=0268-4705&isbn=&volume=35&issue=1&spage=37&pages=37-41&date=2020&title=Current+Opinion+in+Cardiology&atitle=Virtual+reality+in+pediatric+cardiology%3A+Hype+or+hope+for+the+future%3F&aulast=Sacks&pid=%3Cauthor%3ESacks+L.D.%3BAxelrod+D.M.%3C%2Fauthor%3E%3CAN%3E630450871%3C%2FAN%3E%3CDT%3EReview%3C%2FDT%3E)

9. Future perspectives of digital visualization technologies in vascular surgery: Augmented reality, virtual reality and 3D printing. Zukunftsperspektiven digitaler Visualisierungstechnologien in der Gefaschirurgie: Augmented Reality, Virtual Reality und 3-D-Druck <Zukunftsperspektiven digitaler Visualisierungstechnologien in der Gefaschirurgie: Augmented Reality, Virtual Reality und 3-D-Druck.>

Dorweiler B., Vahl C.F., Ghazy A.

Embase

Gefasschirurgie. 24 (7) (pp 531-538), 2019. Date of Publication: 01 Nov 2019.

[Review]

AN: 2002703522

The implementation of digital technology in surgery is one of the key challenges for current and upcoming generations of vascular surgeons. In the context of vascular medicine, augmented reality (AR), virtual reality (VR) and 3D printing represent digital visualization technologies that will increasingly become implemented; however, the potential applications as well as the process of implementation needs to be evaluated and supervised by the vascular scientific community. The key feature and integral element of all three digital technologies is the electronic model (STL file) of the relevant anatomy that can then be visualized in different ways. The current applications of AR/VR include simulation, rehearsal and training and could be demonstrated to increase time efficiency and safety. While the AR technology has already been successfully tested in the operating room (although currently not in routine use due to technical challenges), the applicability of VR in the operating room remains to be evaluated. The 3D printing technology is currently used for simulation, clarification and training purposes as well as in patient education and for generation of patient-specific cutting guides in the operating room.

Copyright © 2019, Springer Medizin Verlag GmbH, ein Teil von Springer Nature.

Status

Embase

Institution

(Dorweiler, Vahl, Ghazy) Sektion Gefaschirurgie, Klinik und Poliklinik fur Herz-, Thorax- und Gefaschirurgie, Universitatsmedizin Mainz, Langenbeckstrase 1, Mainz 55131, Germany

Publisher

Springer Verlag (E-mail: service@springer.de)

Keyword Heading

Augmented reality, Digitalization, Printing, three dimensional, Surgery 4.0, Virtual reality

MeSH Heading

computer simulation; *digital imaging; human; patient education; patient safety; review; simulation training; surgical anatomy; surgical technology; surgical training; *three dimensional printing; time management; vascular surgeon; *vascular surgery; *virtual reality; operating room; *augmented reality.

Candidate Terms

*augmented reality [other term].

Device Index Terms

operating room.

Official Date

computer simulation; *digital imaging; human; patient education; patient safety; Review; simulation training; surgical anatomy; surgical technology; surgical training; *three dimensional printing; time management; vascular surgeon; *vascular surgery; *virtual reality.

Year of Publication

2019

Link to the Ovid Full Text or citation:

[Click here for full text options](https://ovidsp.ovid.com/athens?T=JS&CSC=Y&NEWS=N&PAGE=fulltext&D=emexa&AN=2002703522)

Link to the External Link Resolver:

[SFX](http://sfxeu05.hosted.exlibrisgroup.com/44RCS?sid=OVID:embase&id=pmid:&id=doi:10.1007%2Fs00772-019-00570-x&issn=0948-7034&isbn=&volume=24&issue=7&spage=531&pages=531-538&date=2019&title=Gefasschirurgie&atitle=Zukunftsperspektiven+digitaler+Visualisierungstechnologien+in+der+Gefaschirurgie%3A+Augmented+Reality%2C+Virtual+Reality+und+3-D-Druck&aulast=Dorweiler&pid=%3Cauthor%3EDorweiler+B.%3BVahl+C.F.%3BGhazy+A.%3C%2Fauthor%3E%3CAN%3E2002703522%3C%2FAN%3E%3CDT%3EReview%3C%2FDT%3E)

10. Usefulness of three-dimensional modeling in surgical planning, resident training, and patient education.

Andolfi C., Plana A., Kania P., Banerjee P.P., Small S.

Embase

Journal of Laparoendoscopic and Advanced Surgical Techniques. 27 (5) (pp 512-515), 2017. Date of Publication: May 2017.

[Article]

AN: 616115283

Background: Imaging has a critical impact on surgical decision making and three-dimensional (3D) digital models of patient pathology can now be made commercially. We developed a 3D digital model of a cancer of the head of the pancreas by integrating actual CT data with 3D modeling process. After this process, the virtual pancreatic model was also produced using a high-quality 3D printer.

Patients and Methods: A 56-year-old female with pancreatic head adenocarcinoma presented with biliary obstruction and jaundice. The CT scan showed a borderline resectable tumor with a clear involvement of the gastroduodenal artery but doubtful relationships with the hepatic artery. Our team in collaboration with the Immersive Touch team used multiple series from the CT and segmented the relevant anatomy to understand the physical location of the tumor. An STL file was then developed and printed.

Result(s): Reconstructing and compositing the different series together enhanced the imaging, which allowed clearer observations of the relationship between the mass and the blood vessels, and evidence that the tumor was unresectable. Data files were converted for printing a 100% size rendering model, used for didactic purposes and to discuss with the patient.

Conclusion(s): This study showed that (1) reconstructing enhanced traditional imaging by merging and modeling different series together for a 3D view with diverse angles and transparency, allowing the observation of previously unapparent anatomical details; (2) with this new technology surgeons and residents can preobserve their planned surgical intervention, explore the patient-specific anatomy, and sharpen their procedure choices; (3) high-quality 3D printed models are increasingly useful not only in the clinical realm but also for personalized patient education.

© Copyright 2017, Mary Ann Liebert, Inc. 2017.

PMC Identifier

27813710 [<http://www.ncbi.nlm.nih.gov/pubmed/?term=27813710>]

Status

Embase

Institution

(Andolfi, Plana) Department of Surgery, University of Chicago, Pritzker School of Medicine, 5841 S. Maryland Avenue, Chicago, IL 60637, United States (Kania) ImmersiveTouch, Inc., Chicago, IL, United States

(Banerjee) Center for Simulation, University of Chicago, ImmersiveTouch, Inc., Chicago, IL, United States

(Small) Department of Anesthesia and Critical Care, Center for Simulation, University of Chicago Medicine, Chicago, IL, United States

Publisher

Mary Ann Liebert Inc. (E-mail: info@liebertpub.com)

Keyword Heading

3D imaging, 3D modeling, 3D printing, Augmented reality, Immersive reality, Pancreatic cancer, Surgical planning

MeSH Heading

adult; article; case report; cholestasis; computer assisted tomography; endoscopic retrograde cholangiopancreatography; female; human; jaundice; middle aged; multiple cycle treatment; neoadjuvant chemotherapy; *pancreas adenocarcinoma/dt [Drug Therapy]; *pancreas adenocarcinoma/su [Surgery]; pancreas adenocarcinoma/dt [Drug Therapy]; *patient education; preoperative care; priority journal; *residency education; surgical training; *three dimensional printing; *treatment planning; virtual reality modeling language; antineoplastic agent/dt [Drug Therapy].

Descriptor UI

antineoplastic agent / drug therapy.

Official Date

adult; Article; case report; cholestasis; computer assisted tomography; endoscopic retrograde cholangiopancreatography; female; human; jaundice; middle aged; multiple cycle treatment; neoadjuvant chemotherapy; *pancreas adenocarcinoma / *drug therapy / *surgery; pancreas adenocarcinoma / drug therapy; *patient education; preoperative care; priority journal; *residency education; surgical training; *three dimensional printing; *treatment planning; virtual reality modeling language.

Year of Publication

2017

Link to the Ovid Full Text or citation:

[Click here for full text options](https://ovidsp.ovid.com/athens?T=JS&CSC=Y&NEWS=N&PAGE=fulltext&D=emed18&AN=616115283)

Link to the External Link Resolver:

[SFX](http://sfxeu05.hosted.exlibrisgroup.com/44RCS?sid=OVID:embase&id=pmid:27813710&id=doi:10.1089%2Flap.2016.0421&issn=1092-6429&isbn=&volume=27&issue=5&spage=512&pages=512-515&date=2017&title=Journal+of+Laparoendoscopic+and+Advanced+Surgical+Techniques&atitle=Usefulness+of+three-dimensional+modeling+in+surgical+planning%2C+resident+training%2C+and+patient+education&aulast=Andolfi&pid=%3Cauthor%3EAndolfi+C.%3BPlana+A.%3BKania+P.%3BBanerjee+P.P.%3BSmall+S.%3C%2Fauthor%3E%3CAN%3E616115283%3C%2FAN%3E%3CDT%3EArticle%3C%2FDT%3E)
