# Supplementary material for: Uses of augmented reality in surgical consent and patient education – A systematic review
Source: PLOS Digit Health. 2025 Apr 28;4(4):e0000777. doi: 10.1371/journal.pdig.0000777 (PMC12036893; doi:10.1371/journal.pdig.0000777)
Supplement: Attachment — Submitted filename: letter 29_10_24.docx [file pdig.0000777.s003.docx]

29-10-2024

Dear Editor

Many thanks to yourself and the reviewers for commenting on our manuscript, we have made the recommended minor revisions below and look forward to seeing our work published in the journal.

**Comment: *In the section, "...Each role must be robustly evaluated with high quality clinical research methods to assess benefits and drawbacks. There is currently no classification system for application of AR in surgery."*

*What should a classification system entail?***

This has been discussed in lines 442-451 of the revised manuscript and refers to the level of fidelity i.e. the extent to which the technology represents reality in a similar way to medical simulation.

***What is an example or two as to how an assessment should be performed to measure benefits and drawbacks?***

We have added lines 442-451 to discuss this, the evaluation would involve a detailed description of the technology and use case so that others can reproduce it, a patient reported measure of effectiveness and use of mental effort scores to ensure understanding of information.

Please note some authors are now in different work locations / institutions and titles I have updated this on the cover page too. No change to authorship was made.

Thank you once again for reviewing our article.

Yours sincerely,

Thomas Stringfellow
