# Supplementary material for: Uses of augmented reality in surgical consent and patient education – A systematic review
Source: PLOS Digit Health. 2025 Apr 28;4(4):e0000777. doi: 10.1371/journal.pdig.0000777 (PMC12036893; doi:10.1371/journal.pdig.0000777)
Supplement: Peer review history

# Author comment: Author response to Decision Letter 0

**DOI:** 10.1371/journal.pdig.0000777.r001

19 Aug 2024

# Editor report: Decision Letter 0

**DOI:** 10.1371/journal.pdig.0000777.r002

**Authors:** Alexander Wong, Christopher Wisnik

11 Oct 2024

PDIG-D-24-00320

Uses of augmented reality in surgical consent and patient education – a systematic review.

PLOS Digital Health

Dear Dr. Stringfellow

Thank you for submitting your manuscript to PLOS Digital Health. After careful consideration, we feel that it has merit but does not fully meet PLOS Digital Health's publication criteria as it currently stands. Therefore, we invite you to submit a revised version of the manuscript that addresses the points raised during the review process.

Please submit your revised manuscript within 30 days Nov 10 2024 11:59PM. If you will need more time than this to complete your revisions, please reply to this message or contact the journal office at digitalhealth@plos.org. When you're ready to submit your revision, log on to https://www.editorialmanager.com/pdig/ and select the 'Submissions Needing Revision' folder to locate your manuscript file.

Please include the following items when submitting your revised manuscript:

* A rebuttal letter that responds to each point raised by the editor and reviewer(s). You should upload this letter as a separate file labeled 'Response to Reviewers'.

* A marked-up copy of your manuscript that highlights changes made to the original version. You should upload this as a separate file labeled 'Revised Manuscript with Track Changes'.

* An unmarked version of your revised paper without tracked changes. You should upload this as a separate file labeled 'Manuscript'.

If you would like to make changes to your financial disclosure, please include your updated statement in your cover letter. Guidelines for resubmitting your figure files are available below the reviewer comments at the end of this letter.

We look forward to receiving your revised manuscript.

Kind regards,

Christopher Wisnik

Guest Editor

PLOS Digital Health

Christopher Wisnik

Guest Editor

PLOS Digital Health

Journal Requirements:

Please review your reference list to ensure that it is complete and correct. If you have cited papers that have been retracted, please include the rationale for doing so in the manuscript text, or remove these references and replace them with relevant current references. Any changes to the reference list should be mentioned in the rebuttal letter that accompanies your revised manuscript. If you need to cite a retracted article, indicate the article's retracted status in the References list and also include a citation and full reference for the retraction notice.

Additional Editor Comments (if provided):

In the section, "...Each role must be robustly evaluated with high quality clinical research methods to assess benefits and drawbacks. There is currently no classification system for application of AR in surgery."

What should a classification system entail?

What is an example or two as to how an assessment should be performed to measure benefits and drawbacks?

[Note: HTML markup is below. Please do not edit.]

Reviewers' comments:

[NOTE: If reviewer comments were submitted as an attachment file, they will be attached to this email and accessible via the submission site. Please log into your account, locate the manuscript record, and check for the action link "View Attachments". If this link does not appear, there are no attachment files.]

While revising your submission, please upload your figure files to the Preflight Analysis and Conversion Engine (PACE) digital diagnostic tool, https://pacev2.apexcovantage.com/. PACE helps ensure that figures meet PLOS requirements. To use PACE, you must first register as a user. Registration is free. Then, login and navigate to the UPLOAD tab, where you will find detailed instructions on how to use the tool. If you encounter any issues or have any questions when using PACE, please email PLOS at figures@plos.org. Please note that Supporting Information files do not need this step.

# Author comment: Author response to Decision Letter 1

**DOI:** 10.1371/journal.pdig.0000777.r003

28 Nov 2024

Attachment Submitted filename: letter 29_10_24.docx

# Editor report: Decision Letter 1

**DOI:** 10.1371/journal.pdig.0000777.r004

**Authors:** Daniel A Hashimoto

7 Feb 2025

Uses of augmented reality in surgical consent and patient education – a systematic review.

PDIG-D-24-00320R1

Dear Mr Stringfellow,

We are pleased to inform you that your manuscript 'Uses of augmented reality in surgical consent and patient education – a systematic review.' has been provisionally accepted for publication in PLOS Digital Health.

Before your manuscript can be formally accepted you will need to complete some formatting changes, which you will receive in a follow-up email from a member of our team. 

Please note that your manuscript will not be scheduled for publication until you have made the required changes, so a swift response is appreciated.

IMPORTANT: The editorial review process is now complete. PLOS will only permit corrections to spelling, formatting or significant scientific errors from this point onwards. Requests for major changes, or any which affect the scientific understanding of your work, will cause delays to the publication date of your manuscript.

If your institution or institutions have a press office, please notify them about your upcoming paper to help maximize its impact. If they'll be preparing press materials, please inform our press team as soon as possible -- no later than 48 hours after receiving the formal acceptance. Your manuscript will remain under strict press embargo until 2 pm Eastern Time on the date of publication. For more information, please contact digitalhealth@plos.org.

Thank you again for supporting Open Access publishing; we are looking forward to publishing your work in PLOS Digital Health.

Best regards,

Daniel A Hashimoto, MD, MSTR FACS

Section Editor

PLOS Digital Health

***********************************************************

**Additional Editor Comments (if provided):**

**Reviewer Comments (if any, and for reference):**
